# An evaluation of liquid-based cytology and human papillomavirus testing within the UK cervical cancer screening programme

**DOI:** 10.1038/sj.bjc.6601884

**Published:** 2004-05-25

**Authors:** C Sherlaw-Johnson, Z Philips

**Affiliations:** 1 Clinical Operational Research Unit, University College London, Department of Mathematics, University College London, Gower Street, London WC1E 6BT, UK; 2 School of Economics, University of Nottingham, University Park, Nottingham NG7 2RD, UK

**Keywords:** cervical cancer, screening, human papillomavirus, liquid-based cytology, cost-effectiveness

## Abstract

The aim of this study is to evaluate different options for introducing liquid-based cytology (LBC) and human papillomavirus (HPV) testing into the UK cervical cancer screening programme. These include options that incorporate HPV testing either as a triage for mild and borderline smear abnormalities or as a primary screening test. Outcomes include the predicted impact on resource use, total cost, life years and cost–effectiveness. Extensive sensitivity analysis has been carried out to explore the importance of the uncertainty associated with disease natural history and the impact of screening. Under baseline assumptions, the cost–effectiveness of different options for introducing LBC appears favourable, and these results are consistent under a range of assumptions for its impact on the diagnostic effectiveness of cytology. However, if we assume a higher marginal cost of LBC in comparison to conventional methods, primary smear testing options are predicted to be more cost-effective without LBC. Combined LBC primary smear and HPV testing with a 5-year interval is similar in both cost and effectiveness to the other 3-yearly options of primary smear testing or primary HPV testing alone. However, both primary HPV testing and combined options would give rise to a far greater risk of inappropriate colposcopy throughout a woman's lifetime.

Liquid-based technology has been developed for the purpose of improving the preparation of cervical smear samples, and a range of different methods are available which include AutoCytePrep, Easy Prep, Cytoscreen and Thinprep. Each involves the preparation of potentially better and more representative smear samples for cytological examination than with conventional means, with a consequent reduction in the number of inadequate specimens and an increase in sensitivity. In practice, however, the actual impact on the performance of cytology is still a matter of some debate since different studies have reported very different findings. Currently, in the UK, around 10% of all smear samples are classified as inadequate, and the recent Pilot Site evaluation of liquid-based cytology (LBC) in the UK found that the new technology reduced this figure to 2% (Moss *et al*, 2002). However, there are other studies that have reported very little impact (Dupree *et al*, 1998; Diaz-Rosario and Kabawat, 1999). Similarly, with smear test sensitivity, some studies have demonstrated improvements (Nanda *et al*, 2000), while others have found little (Moss *et al*, 2002; Coste *et al*, 2003).

As human papillomavirus (HPV) testing technology has developed, the inclusion of HPV testing within cervical screening programmes has been suggested, either as a triage for women presenting with borderline and mildly abnormal smears (Cox *et al*, 1995) or as a primary screen (Cuzick *et al*, 1999b; Cuzick *et al*, 2003). It has also been suggested that, with HPV testing, routine screening intervals could be lengthened without compromising effectiveness. Under conventional cytology, a separate smear sample needs to be taken for the HPV test, whereas with liquid-based preparations, the same smear sample could be used for both cytology and the HPV test (Ferenczy and Franco, 1997). This may make the introduction of HPV testing more viable, with important implications for cost and convenience. It may also mean that screening options that combine smear and HPV testing in primary screening become worthwhile (Meijer *et al*, 1997).

The purpose of this study is to evaluate different options for introducing LBC within the UK, in terms of the impact on resource use, cost, effectiveness and overall cost–effectiveness. The merits of liquid-based approaches are also compared with other changes that could be made to the screening programme keeping conventional techniques, including the introduction of HPV testing. This is the only study that compares such a range of options simultaneously. The study does not investigate one specific liquid-based method but a range of approaches defined by different characteristics of cost and impact. We have used a mathematical modelling approach based upon methods developed by the authors in previous studies (Jenkins *et al*, 1996; Sherlaw-Johnson *et al*, 1999). Mathematical modelling has value in this context since it provides a method for assessing a wide range of screening options relatively quickly.

## METHODS

### Mathematical model

The main elements of the model are the clinical course of cervical disease and resulting mortality, age-related mortality from other causes, the accuracy of screening tests and the screening policy. The model is stochastic in that we use methods from probability theory to represent the considerable variability in possible outcomes.

**Figure 1:**
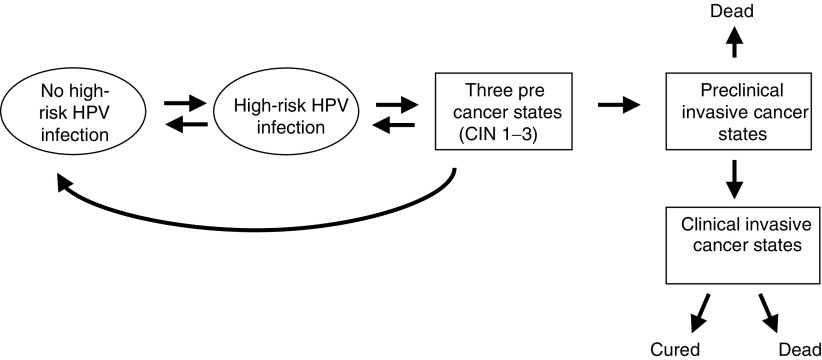
Model representation of disease natural history.

The clinical course of the disease and the development of invasive cancer are represented by a sequence of transitions through cervical precancer and stages of preclinical and clinically invasive cancer (Figure 1

). Precancers are assumed to be preceded by high-risk HPV infection (most commonly, types 16, 18, 31 and 33), and are divided into three separate states corresponding to the three grades of cervical intraepithelial neoplasia (CIN 1–3). The cancer states correspond to the International Federation of Gynecology and Obstetrics (FIGO) classification stages I–IV. Although not indicated in Figure 1, account is also taken of deaths from other causes, which, after correcting for age and sex, are assumed to be independent of the stage of cervical disease. This disease progression model is stochastic in that the transitions between states are assumed to be a chance process with a specified probability assigned to each transition. Starting with a woman aged 15 years who is free from disease, the model predicts the probability of being in each disease category at all subsequent ages. If the woman is screened then the chances of detecting any precancerous lesion, or preclinical cancer will depend on the accuracy of the screening test. Successful detection and treatment of precancerous lesions are modelled by assuming that women revert to being disease free, with a prespecified proportion of HPV infections regressing simultaneously. Screening occurs at different ages depending upon the programme and the policy for the follow-up of abnormal results. Under the baseline assumptions, successive HPV tests on the same women are assumed to be independent, but the possibility that HPV remains undetectable in certain women over a series of tests is also assessed.

Parameter values for the model have been derived from existing data sources. However, there is much uncertainty relating to these values with several sources reporting very different results. Owing to this uncertainty, we have undertaken extensive sensitivity analysis to investigate how it affects outcomes.

### Natural history assumptions

Structurally, the disease progression model is a continuous time Markov Process governed by instantaneous hazard rates between each state (Ross, 1993). The implications of the chosen rate parameters in terms of more easily interpreted annual progression rates are shown in Table 1

**Table 1:** Assumptions concerning the natural history of HPV and cervical precancers (alternative assumptions in parentheses)

**Parameter**	**Value**	**Reference**
Percentage of women contracting high-risk HPV infections over a given year	30% declining to 0% with age (20% declining to 0%, 40% declining to 0%)	*Prevalence data*: Melkert *et al* (1993); Cuzick *et al* (1999a)
Mean persistence of HPV infections	1 year increasing to 17 years with age (1.5 increasing to 28)	*Incidence and regression*: Hildesheim *et al* (1994); Moscicki *et al* (1993); Ho *et al* (1998)
		
Percentage of women contracting CIN over a given year in the absence of high-risk HPV	0% for all ages (0.8% decreasing to 0% with age)	
Percentage of high-risk HPV infections progressing to CIN over a year	19% increasing to 24% with age	*Progression and regression*: Ho *et al* (1998); Koutsky *et al* (1992)
Percentage of CIN 1 lesions progressing over a year	13% increasing to 17% with age (19% increasing to 24% with age)	*Cancer incidence data*: Office for National Statistics (2003)
Percentage of CIN 2 lesions progressing over a year	17%	
Percentage of CIN 3 lesions progressing over a year	0.7%	*Screening and colposcopy data*: Department of Health (2001)
Annual regression of CIN 1 lesions	70% declining to 20% with age	
Annual regression of CIN 2/3 lesions	7% declining to 2% with age	
		
Mean duration of preclinical invasive cancer stages	Stage 1: 5 months; stage 2: 1.5 months; stage 3: 1 months; stage 4: 20 days	
Stage distribution of new clinical cancers	Stage 1: 40%; stage 2: 31%; stage 3: 21%; stage 4: 8%	
Proportion of stage 1 cancers cured	95% decreasing to 70% with age (89% decreasing to 65%)	Meanwell *et al* (1988); Kosary (1994); Office for National Statistics (1999)
Proportion of stage 2 cancers cured	65% decreasing to 45% (61% decreasing to 42%)	
Proportion of stage 3 cancers cured	45% decreasing to 30% (35% decreasing to 26%)	
Proportion of stage 4 cancers cured	20% decreasing to 10% (14% decreasing to 7%)	
Age-related mortality from other causes	England and Wales, female life tables	Government Actuary's Department, 1996

CIN=cervical intraepithelial neoplasia; HPV=human papillomavirus.

, together with references to the studies from which they have been derived. (Detailed assumptions are available from the authors by request.) For many disease progression parameters, direct estimates are not available from the literature. Values have thus been inferred from observed data such as the prevalence of HPV infection, the overall incidence of invasive cancer and the prevalence of precancers detected among a screened population. This method has not generated a unique set of parameters, but rather a number of plausible sets that have all been used by the model to explore the consequences of different disease progression scenarios. These scenarios are defined by different rates of prevalence and persistence of HPV infection, and the incidence of cervical cancer if the current population were not screened. The range of values encompassed by these scenarios is also shown in Table 1. The baseline scenario was validated against the age-related incidence of invasive cancer in the UK (Office for National Statistics, 2003).

### Diagnosis

Other parameters used in the model that relate to diagnostic accuracy and the impact of LBC are shown in Table 2

**Table 2:** Assumptions concerning the performance of screening tests (alternative assumptions in parentheses)

**Parameter**	**Value**	**Reference**
Probability of a borderline or mild smear result given the underlying histology	Normal 2.8% (2.0%)	
	CIN 1 53%	
	CIN 2 23% (30%)	
	CIN 3 24% (20%)	*Baseline*: Cuzick *et al* (1995)
	Invasive cancer 40%	*Invasive cancer*: inferred from Department of Health (2001)
Probability of a moderate or severe smear result given the underlying histology	Normal 0.6% (0%)	*Range*: Jenkins *et al* (1998)
	CIN 1 14%	
	CIN 2 18% (40%)	
	CIN 3 41% (60%)	
	Invasive cancer 60%	
Sensitivity of HPV test at detecting high-grade CIN	88% (65%, 95%)	*Baseline*: Schiffman *et al* (2000). *Range*: Jenkins *et al* (1998)
Proportion of inadequate smear results	10% (5%)	Department of Health (2001)
Impact of liquid-based cytology on smear test sensitivity	No increase (40% increase in detected precancers)	*Baseline*: Moss *et al* (2002) *Alternative*: Payne *et al* (2000)
Proportion of inadequate smear test results with liquid-based cytology	5% (2%, 10%)	*Baseline and upper alternative*: Payne *et al* (2000); McCrory *et al* (1999)
		*Lower alternative*: Moss *et al* (2002)

. There is also much uncertainty relating to these values: several studies and different techniques report very different results. Owing to this uncertainty, we have investigated the consequences of using a range of values for the same parameter (also shown in Table 2).

### Costs

Costing was undertaken from the perspective of the health care provider. All costs are represented as 2001 values and are summarised in Table 3

**Table 3:** Unit cost assumptions for screening strategies and follow-up

**Item**	**Cost (£) (ranges in parentheses)**	**Source**
*Conventional screening*		*Takers and administration costs*: Netten *et al* (2001); CIPFA (2002)
Smear test	20.76 (19.69–21.84)	
HPV test	22.29 (20.29–25.29)	*Laboratory and HPV costs*: Laboratory Accounts
*Primary smear testing with LBC*		*LBC costs*: Payne *et al*, 2000.
Smear test	20.00 (18.93–23.60)	
Marginal cost of HPV test	11.79 (9.79–14.79)	
*Primary HPV testing with LBC*		
HPV test	20.21 (18.21–23.21)	
Marginal cost of smear test	11.58 (10.51–15.18)	
		
Colposcopy	71.19 (56.95–85.43)	
Punch biopsy	59.02 (47.22–70.82)	Wolstenholme 2002,
CIN I	311 (249–373)	
CIN II	343 (274–412)	
CIN III	369 (295–443)	
		
Stage 1 invasive cancer	9772 (7818–11 726)	
Stage 2 invasive cancer	16 097 (12 878–19 316)	Wolstenholme and Whynes 1998
Stage 3 invasive cancer	15 991 (12 793–19 189)	
Stage 4 invasive cancer	17 020 (13 616–20 424)	

. The cost of conventional smear taking is derived from primary resource utilisation data. In total, 66 GP practices in the UK completed a questionnaire regarding the time spent on smear taking, administration and the personnel involved. On average, 85% of smears were taken by a practice nurse, 14% by the GP and 1% by hospital or family planning personnel. The average time spent on a smear (including preparation) was between 14 and 18 min. Staff time was valued using standard sources (Netten *et al*, 2001; CIPFA, 2002). As a base case assumption, the time involved in smear taking using LBC was assumed to be 4 min less than with conventional methods. This assumption was varied in sensitivity analysis. The smear taker was assumed to follow the same distribution as for conventional methods. Primary care administration was found to take on average 3.7 h per week and each practice in the survey performed approximately 12.5 smear tests per week. We had no grounds to assume that administration costs would differ between conventional and LBC methods. NHS administration, including GP target payments, are excluded from these estimates. Laboratory costs for conventional cytology are based on an audit of the City Hospital cytology laboratory in Nottingham, and include staff costs, equipment (including maintenance), transport and consumables. Under LBC, staffing within the laboratory was assumed to be equal to those of conventional cytology. Equipment, consumables and maintenance costs for LBC methods are taken from reported data (Payne *et al*, 2000). Human papillomavirus test costs include staff time, equipment (inc. maintenance) and consumables. Human papillomavirus test transportation costs are not included in this estimate. Cervical intraepithelial neoplasia and invasive cancer treatment costs are taken from detailed cost of illness studies (Wolstenholme, 2002; Wolstenholme and Whynes, 1998). Further details of resource use and costing methods are available on request.

### Screening

The screening options evaluated in this study include:

screening all women from age 21 to 64 years. Mildly abnormal and borderline results during routine screening are followed up according to UK recommendations for repeat cytology;
routine screening as in (i) with HPV testing as a triage for mild and borderline smear results during routine screening. If the HPV test is positive for high-risk types, women are referred for colposcopy, otherwise they have repeat cytology as in (i);
option (i) until the age of 30 years after which routine smear testing is replaced by primary HPV testing with cytology as a triage for women found to be positive for high-risk HPV types. If the cytological result is abnormal, women are referred for colposcopy, otherwise they have a follow-up HPV test in 6 months. If this repeat HPV test is negative, women revert to routine screening;
option (i) until the age of 30 years, after which primary smear testing is combined with HPV testing. Women with moderate or severe smear abnormalities are referred for colposcopy. Women with mild or borderline smear results, or a negative smear and a positive HPV test undergo repeat combined testing at 6-month intervals. Women revert to routine screening after a combination of a clear smear test and negative HPV result.

Option (i) approximates to current recommended screening policy in the UK. The other options are based upon suggested strategies for including HPV testing as a triage (Beral and Day, 1992; Cox *et al*, 1995), as a primary screen with cytology as a triage (Cuzick *et al*, 2003), or combined with cytology as a primary screen (Meijer *et al*, 1997; Cuzick *et al*, 1999b).

Each screening option was evaluated with 3- and 5-year routine intervals and both with and without LBC. We have assumed that 85% of eligible women throughout the population are screened, which is similar to current coverage rates in the UK (Department of Health, 2001), and that this is the same for all ages at which women are screened.

### Outcomes

Outcomes are presented as average and incremental costs and life years gained over the lifetime of women aged 15 years, and who are exposed to each screening option throughout (although not necessarily attending). In line with current UK recommendations, future costs and life years were discounted at 3.5% per annum, along with the consequences of no discounting and of discounting costs and life years at 6% per annum (National Institute for Clinical Excellence, 2003).

Incremental cost–effectiveness ratios are defined as the ratio of the difference between cost and outcome of moving from one option to the next in order of increasing cost. In calculating incremental cost–effectiveness ratios, all dominated and extendedly dominated options are excluded. An option is ‘dominated’ if there is another option which is both cheaper and more effective. An option is ‘extended dominated’ if it is not dominated by a single other option, but there is a combination of two options that is both cheaper and more effective (Drummond *et al*, 1997). Options that are neither dominated nor extended dominated define the ‘efficiency frontier’.

## RESULTS

### Costs and effectiveness

With the baseline assumptions, average lifetime costs and life expectancy from age 15 years are shown in Table 4

**Table 4:** Outcomes under baseline assumptions. Incremental cost–effectiveness ratios represent the cost for every year of life gained by adopting the more expensive option in favour of the next cheapest, nondominated option

**Options in order of increasing cost**	**Average lifetime cost of screening and treatment (discounted at 3.5% p.a.)**	**Average life expectancy from age 15 years (discounted at 3.5% p.a.)**	**Incremental cost–effectiveness ratios**	**Total deaths averted**	**CIN 2/3 detected per lifetime**	**Inappropriate referrals for colposcopy per lifetime**
No screening	£86.14	25.292	—	—	—	—
Repeat cytology with LBC, 5 years	£159.79	25.316	ED	64.89%	0.152	0.084
HPV triage with LBC, 5 years	£162.54	25.317	£3,067	67.63%	0.157	0.099
Repeat cytology without LBC, 5 years	£166.06	25.316	D	64.89%	0.152	0.083
Primary HPV with LBC, 5 years	£169.50	25.319	£3,720	71.29%	0.156	0.390
HPV triage without LBC, 5 years	£170.38	25.317	D	66.72%	0.157	0.099
Primary HPV without LBC, 5 years	£187.72	25.318	D	68.55%	0.150	0.367
Repeat cytology with LBC, 3 years	£214.59	25.320	ED	73.12%	0.170	0.132
HPV triage with LBC, 3 years	£219.23	25.321	ED	74.95%	0.175	0.158
Combined cytology and HPV with LBC, 5 years	£219.50	25.321	£22,628	77.69%	0.166	0.515
Repeat cytology without LBC, 3 years	£224.13	25.320	D	72.20%	0.170	0.131
Primary HPV with LBC, 3 years	£227.87	25.321	ED	74.03%	0.153	0.613
HPV triage without LBC, 3 years	£231.17	25.321	D	74.03%	0.175	0.157
Primary HPV without LBC, 3 years	£256.01	25.320	D	70.38%	0.147	0.574
Combined cytology and HPV with LBC, 3 years	£309.58	25.323	£37,846	80.43%	0.160	0.810

**Figure 2:**
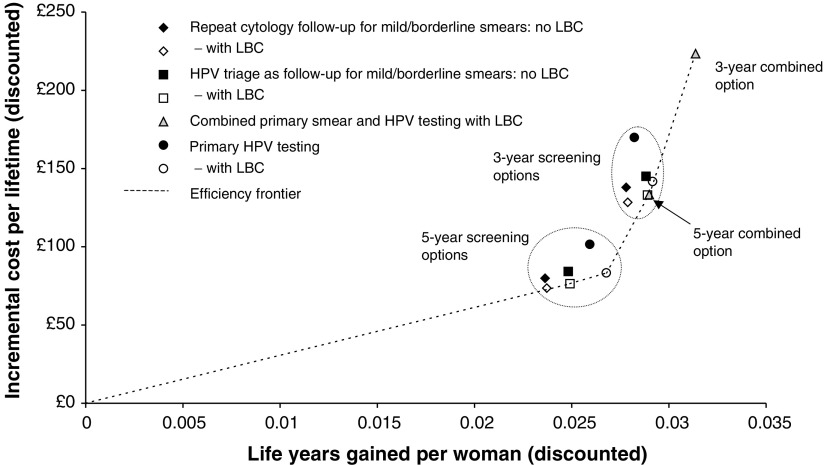
Incremental costs and life years gained of different options in comparison to no screening. Costs and life years are discounted at 3.5% per annum.

D=dominated; ED=extended dominated; LBC=liquid-based cytology; HPV=human papillomavirus.

, together with the corresponding incremental cost–effectiveness ratios. All options that do not involve LBC are dominated. Incremental costs and life years saved for each option are also illustrated in Figure 2

, together with the efficiency frontier. Apart from the two combined options, the 5- and 3-yearly screening options form two distinct groups, with the 5-year combined option having similar results to the other 3-year strategies. All the options that include LBC are either on, or very close to the efficiency frontier, except, perhaps for the 3-year repeat cytology strategy. Figure 2 also indicates that the main impact of LBC is a cost reduction as opposed to improved effectiveness.

Other outcomes are shown in Table 4, including the percentage of deaths due to cervical cancer that are averted by screening and the lifetime rate of detection of high-grade CIN. The final column shows the lifetime risk of an inappropriate referral for colposcopy, with a referral being ‘inappropriate’ if there are no precancerous or cancerous lesions present. The risk of inappropriate referral is highest under the primary HPV and combined options where it can be over 50%, while under primary smear testing, the risk is no higher than 16%.

### Sensitivity analysis

The results of one-way sensitivity on the impact of LBC and discounting are shown in Table 5

**Table 5:** Incremental costs per life year gained under different assumptions relating to the performance and cost of LBC

**Options in order of increasing cost except where specified**	**Baseline**	**No impact on inadequates**	**Inadequates reduced to 2% of all smears**	**Marginal cost of LBC=£3.60^c^**	**No discounting**	**Discounting costs and life years at 6% p.a**
No screening	—	—	—	—	—	—
Repeat cytology, LBC, 5-yearly	ED	ED	ED	D	ED	ED
HPV Triage, LBC, 5-yearly	£3067	ED	£2990	D	£683	ED
Repeat cytology, no LBC, 5-yearly	D	D	D	ED	D	D
Primary HPV, LBC, 5-yearly	£3720	£3167	£4027	£3368	£2011	£6588
HPV Triage, no LBC, 5-yearly	D	D	D	ED	D^a^	D
Primary HPV, no LBC, 5-yearly	D	D	D	D	D	D
Repeat cytology, LBC, 3-yearly	ED	ED	ED	D	ED	D
HPV Triage, LBC, 3-yearly	ED	ED	ED	D	ED	ED
Combined, LBC, 5-yearly	£22 628	£18 486	D	ED	£9309	ED^b^
Repeat cytology, no LBC, 3-yearly	D	D^b^	D	ED	D^a^	D
Primary HPV, LBC, 3-yearly	ED	D	£24 149	£26 242	D	£34 093^a^
HPV Triage, no LBC, 3-yearly	D	D	D	ED	D^a^	D
Primary HPV, no LBC, 3-yearly	D	D	D	D	D	D
Combined, LBC, 3-yearly	£34,686	£38 910	£46 569	£44 362	£21 115	£68 198

Monetary values represent the cost for every year of life saved by adopting the more expensive option in favour of the next cheapest, nondominated option. D=dominated; ED=extended dominated; LBC=liquid-based cytology; HPV=human papillomavirus.

a This option is cheaper than the one above.

b This option is cheaper than the two above.

c Under this assumption, the order of the LBC and non-LBC options for each of the repeat cytology and HPV triage options are reversed.

. The options are again listed in order of increasing cost, except where indicated. Even if LBC has no impact on the number of inadequate smears, all the conventional options are still dominated. Although not indicated by this table, the options that involve primary smear testing and LBC with HPV triage consistently lie on, or close to the efficiency frontier. If we assume a high marginal cost of LBC over conventional smears, options that involve conventional methods are predicted to be cheaper for primary smear testing, but not for primary HPV testing.

Figure 3

**Figure 3:**
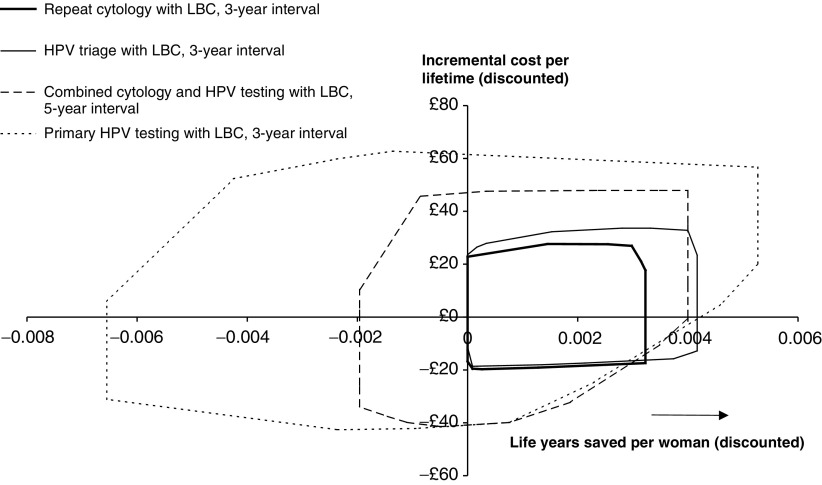
Incremental costs and life years gained of different options in comparison to the baseline option of 3-year screening with repeat cytology following mild or borderline smear results and no LBC. Costs and life years are discounted at 3.5% per annum. The areas represent the range of outcomes from all possible combinations of alternative assumptions used in the sensitivity analysis, except discounting.

shows the results of multiway sensitivity analysis on the impact of four options for using LBC in comparison to 3-year screening with repeat cytological follow-up and no LBC. Since this latter strategy is an approximation to current recommended practice in the UK, the graph shows the likely impact of changing policy. This multiway analysis includes all possible combinations of alternative assumptions as specified in Tables 1, 2 and 3 and so represents the full range of uncertainty under the baseline discount rates. The two primary smear testing options are consistently predicted to either match or increase effectiveness. The less-precise forecasts for the combined, and, in particular, the primary HPV testing options are consequences of the uncertainty regarding the accuracy and prevalence of the HPV test. Decreased effectiveness will occur with combinations of a test that is less accurate than the baseline assumption, an assumption that some HPV remains consistently difficult to detect and an assumed higher prevalence of infection in older women.

## DISCUSSION

The purpose of this study has been to evaluate and compare options for introducing LBC and/or HPV testing into cervical cancer screening programmes. All the screening options have been chosen to reflect those that have been previously proposed or evaluated. However, this is the only study that has evaluated all screening options in combination.

Under baseline assumptions, the cost–effectiveness of different options for introducing LBC appears favourable, and these results are consistent under a range of assumptions for its impact on the diagnostic effectiveness of cytology. However, the marginal cost of LBC does affect outcomes. If we assume a higher marginal cost of LBC in comparison to conventional methods, primary smear testing options are predicted to be more cost-effective without LBC. The cost–effectiveness of primary HPV testing with LBC appears less sensitive to changes in the LBC marginal cost, but this is because in our one-way sensitivity analysis, variability in cost is only associated with the cytology laboratory costs and not HPV costs. Combined primary smear and HPV testing with a 5-year interval is similar in both cost and effectiveness to the other 3-yearly options. However, both the primary HPV testing and combined options would give rise to a far greater risk of inappropriate colposcopy throughout a woman's lifetime.

Adding LBC to the current programme is predicted to match or improve effectiveness under the full range of assumptions used for sensitivity analysis, and, likewise, if it was further modified by adding HPV testing as a triage for mild or borderline smear abnormalities. Changing to the combined smear and HPV testing option and raising the screening interval to 5 years, could improve effectiveness, similarly if the programme is changed to primary HPV testing. However, under these options, the predicted impact is less robust to the assumptions we have made: their effectiveness being very sensitive to the relative diagnostic accuracy of the HPV test in relation to cytology, HPV prevalence and whether there is any consistent difficulty in detecting HPV infections in some women.

Since a modelling approach has been used, there has been scope for analysing a wide range of options. Several different scenarios have been analysed to allow for the uncertainty regarding the model parameters. This uncertainty not only reflects the range of estimates reported in the scientific literature, but also the imprecise knowledge regarding the natural history and age-related prevalence of HPV infections and cervical precancers both now and in the future. Evaluating different scenarios has enabled us to investigate how robust predicted outcomes are to changes in model assumptions, to identify areas of uncertainty that have a large influence on outcome, and identify circumstances under which particular options are less favourable.

The costs used in the model are based on best estimates currently available, although no account has been taken of the training and transfer costs associated with moving from the current screening programme to one including LBC and/or HPV testing and neither have transportation costs for HPV samples been included. It is recognised that the prospective resource utilisation data come from a relatively small number of practices in the UK and the cost data for LBC are based largely on assumption.

Previous studies have analysed the impact of LBC on cervical screening programmes (McCrory *et al*, 1999; Payne *et al*, 2000; Kim *et al*, 2002; Moss *et al*, 2002). All are based wholly, or partly on mathematical modelling, but none have investigated the whole range of options in this study together. All studies conclude that introducing LBC within a primary smear testing programme could be cost-effective with (Kim *et al* 2002), or without (McCrory *et al*, 1999; Payne *et al*, 2000; Moss *et al*, 2002) HPV testing as a triage. They all predict improvements in effectiveness, but the impact on cost depends on the assumed marginal cost for the new technology. Of these, the lowest marginal cost was calculated by the UK Cervical Screening Pilot Site study, which is the only one to predict overall cost savings. This study also found that LBC reduces the number of inadequate smears by about 80% yet has little impact on test sensitivity. Three studies have investigated combined smear and HPV testing and HPV testing alone as primary screening methods, but without LBC (Van Ballegooijen *et al*, 1997; Cuzick *et al*, 1999b; Mandelblatt *et al*, 2002). The latter of these only compared 2- and 3-year screening intervals, yet found a combined test to be feasible if extended to an older age group. The two former studies, as with this study, found combined screening options at 3- and 5-year intervals to be more costly yet more effective than 3-year smear testing, much of the extra cost being due to screening and surveillance. Also, similar to our own findings, the relative impact of primary HPV testing was less robust to different versions of their model and so difficult to determine.

The mathematical model is a tool for making the best use of the available information, yet the results cannot be treated as definitive. There are further factors that have not been investigated which could influence the outcomes. Women are assumed to be either always compliant or not, and no account has been taken of how compliance, and hence effectiveness, could be improved by reducing the number of tests a woman is required to attend, for example, by extending the screening interval, combining smear and HPV tests and reducing the number of inadequate smears. The performance of screening tests has also assumed to be the same for all ages, although there is evidence that cytological accuracy declines among older women (Mandelblatt *et al*, 1991). There is much controversy over appropriate discount rates, and incremental cost–effectiveness ratios can only be interpreted against the rates that are chosen. However, alternative views of discounting have little impact on how different options stand in relation to each other.

The aim of this study was to evaluate screening in the context of the UK, and care would need to be taken before translating its results to other settings. International comparisons of cost–effectiveness are hampered by differences in health systems, resource and cost structures, chosen comparators and recommended methods for evaluating technologies. Guidelines on appropriate discount rates differ between countries; in the United States for example, the current recommendations are 3% for both costs and life years (Weinstein *et al*, 1996). Perhaps more importantly, at 9–10%, the proportion of smears classified as inadequate is particularly high in the UK in comparison to other developed countries, and, as a result, any reduction in this proportion due to the introduction of liquid-based technology may be of greater benefit than elsewhere.

Since cost and life years saved are not the only outcomes of importance, other measures could come to the fore in the decision making process. In terms of the psychological impact on women, high false positive rates for referral to colposcopy may count against the acceptability of primary HPV testing and combined primary smear and HPV testing. This may also be an unnecessary use of expensive resources. The HART study, however, suggested that this problem could be overcome in primary HPV testing by annual monitoring of women who are HPV positive with borderline or negative cytology (Cuzick *et al*, 2003). Also, for women being screened, lower numbers of inadequate smears would be desirable, and evidence suggests such reductions occur with LBC without compromising sensitivity (Moss *et al*, 2002).
